# Quantitative Electromyographic Analysis of Reaction Time to External Auditory Stimuli in *Drug-Naïve* Parkinson's Disease

**DOI:** 10.1155/2014/848035

**Published:** 2014-03-02

**Authors:** Do-Young Kwon, Byung Kyu Park, Ji Won Kim, Gwang-Moon Eom, Junghwa Hong, Seong-Beom Koh, Kun-Woo Park

**Affiliations:** ^1^Department of Neurology, Korea University College of Medicine, Ansan Hospital, 516 Gojan-1-dong, Danwon-gu, Ansan-city, Gyeonggi-do 425-707, Republic of Korea; ^2^Department of Physical Medicine & Rehabilitation, Korea University College of Medicine, Ansan Hospital, 516 Gojan-1-dong, Danwon-gu, Ansan-city, Gyeonggi-do 425-707, Republic of Korea; ^3^School of Biomedical Engineering, Konkuk University, Seoul, Republic of Korea; ^4^Department of Control and Instrumentation Engineering, Korea University, Seoul, Republic of Korea

## Abstract

Evaluation of motor symptoms in Parkinson's disease (PD) is still based on clinical rating scales by clinicians. Reaction time (RT) is the time interval between a specific stimulus and the start of muscle response. The aim of this study was to identify the characteristics of RT responses in PD patients using electromyography (EMG) and to elucidate the relationship between RT and clinical features of PD. The EMG activity of 31 PD patients was recorded during isometric muscle contraction. RT was defined as the time latency between an auditory beep and responsive EMG activity. PD patients demonstrated significant delays in both initiation and termination of muscle contraction compared with controls. Cardinal motor symptoms of PD were closely correlated with RT. RT was longer in more-affected side and in more-advanced PD stages. Frontal cognitive function, which is indicative of motor programming and movement regulation and perseveration, was also closely related with RT. In conclusion, greater RT is the characteristic motor features of PD and it could be used as a sensitive tool for motor function assessment in PD patients. Further investigations are required to clarify the clinical impact of the RT on the activity of daily living of patients with PD.

## 1. Introduction

Progressive degeneration of the nigrostriatal pathway results in a deficit of dopaminergic neurons and an imbalance in the corticobasal ganglia-thalamocortical circuit, causing motor dysfunctions in PD [1, 2]. Objective measurement of PD symptoms is problematic, because the PD motor symptoms are usually quantified clinically with UPDRS scores that rely on a physician's subjective scoring. Reaction time (RT) is the time interval between a specific stimulus and the reaction to it. RT is occupied by a train of processes or stages, which are composed of mental processing and motor reaction. RT measurement has been shown by several investigators to be a useful tool for the assessment of motor response and cognitive function evaluation [3–8]. Previous RT studies using electromyography (EMG) in stroke patients demonstrated a significantly longer RT for the initiation and termination of muscle responses [9, 10]. Because cardinal motor dysfunctions in PD are under the control of basal ganglia, defective basal ganglia function might be reflected in RT values; however, the correlation between RT and clinical symptoms and the underlying mechanism is unclear [3, 5, 7, 11–15]. Also, it is assumed that the presence of nonmotor symptoms in PD possibly has an adverse effect on motor functions [16–18]. The primary purpose of this study was to describe the relationship between RT [RTi (delay in initiation of muscle contraction) and RTt (delay in termination of muscle contraction)] and clinically measured motor/nonmotor scores in untreated *de novo* PD patients. In this study, we quantified motor functions by RT measurements based on EMG signals of PD. Furthermore, we examined whether nonmotor PD symptoms had an effect on RT by comparing clinical measurements and acquired EMG signals. The second aim of this study was to evaluate if RT varied according to the PD stages.

## 2. Methods

### 2.1. Subjects and Methods

We recruited thirty-one untreated *de novo* PD patients from an outpatient Parkinson's disease and movement disorder clinic of an academic medical center. Clinical diagnosis of PD was made according to the clinical criteria described by the United Kingdom Parkinson's Disease Society Brain Bank [19]. Age-matched healthy control subjects (*n* = 15) were recruited from among respondents to an advertisement in the hospital. Subjects having significant comorbid systemic disorders, previous motor defects, or taking medications that could affect cognitive function were excluded from the study. Dementia patients who could not follow a simple three-step command were also excluded. Written informed consent for study participation was obtained from all subjects. This study was approved by the Institutional Ethics Review Board. All PD patients underwent magnetic resonance imaging of the brain to rule out symptomatic organic lesions and nerve conduction study was performed to exclude peripheral neuropathy.

Drug-naïve *de novo* PD patients were enrolled in this study because clinically measured scores can be affected by dopaminergic drugs [20]. Motor function was assessed clinically using UPDRS part III [21] and the H&Y stages [22]. Patients were subclassified according to the Hoehn and Yahr (H&Y) stage: Hoehn and Yahr (H&Y) stage 1, H&Y stage 2, and H&Y stage greater than 2.5. Each side of the patient was evaluated to confirm which the more- and less-affected sides were according to the UPDRS score. Motor function assessment was performed within 1 hour before the measurement of RT to avoid possible discrepancies due to diurnal symptom fluctuations. To assess neuropsychiatric function of the patients, expert psychologist evaluated detailed neuropsychological battery [23]; patients who scored below the 9% of normal values were considered to be abnormal group. The age of the 31 *de novo* PD patients (19 men and 12 women) ranged between 34.1 and 79.7 years (median 67.6 years). The healthy controls (5 men and 10 women) ranged in age from 53 to 73 years (median 64.0 years). All participants were right hander. There were no statistical differences between patients and controls in terms of age or sex distribution. The H&Y stage of all participants with PD was 2.02 (range: 1–4) and the average UPDRS part III (motor function) score was 19.13 (total 108).

### 2.2. RT Measurements Using Surface EMG

**Figure 1:**
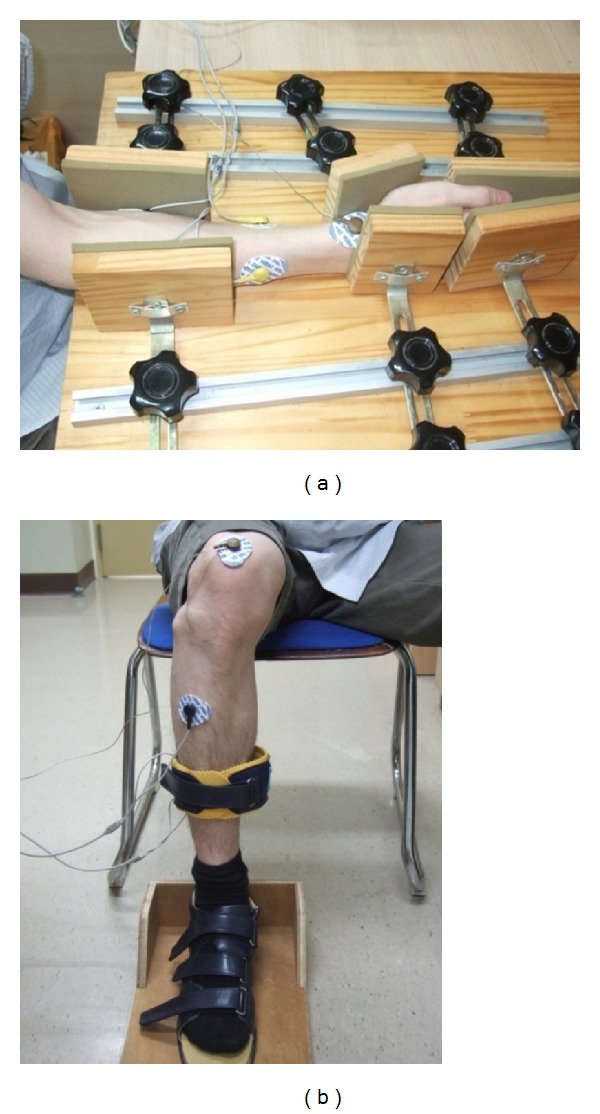
(a) The apparatus for fastening the forearm during isometric wrist flexion and extension. (b) The apparatus that made for foot and leg fixation for EMG recording during isometric ankle dorsiflexion. The sole of the shoe is attached to the board for isometric exercise.

RT values (RTi and RTt) were measured from both upper and lower extremities using surface EMG during isometric muscle contraction. We used a 4-channel EMG machine and conductive adhesive foam electrocardiogram-disposable Ag/AgCl transcutaneous surface recording electrodes (Meditrace 200, Tyco healthcare, USA) for signal recording. A subject was seated on a chair and the extremities were placed in an apparatus using established methods to stabilize and fix during isometric muscle contraction (Figures 1(a) and 1(b)) [9, 10]. For the upper extremities, the surface electrode was placed over the flexor carpi radialis and the extensor carpi radialis (Figure 1(a)), and for the lower extremities, specially-designed shoe-type apparatus with the sole attached to the wooden board was worn to record isometric ankle dorsiflexion (Figure 1(b)). The surface electrode was placed over the belly of the tibialis anterior muscle. Subjects were instructed to contract the corresponding muscle quickly and strongly against the backboard of the apparatus as soon as possible when they heard an audible beep and then terminate muscle contraction as quickly and completely as possible when they recognized that the beep terminated. The auditory beep signal consisted of a total of six audible beeps with three beeps of 3 seconds and three beeps of 6 seconds presented in a random order to minimize the participant's anticipation. The time between beeps was also randomized by the computer to be either 3, 4, or 5 seconds to minimize anticipation.

**Figure 2:**
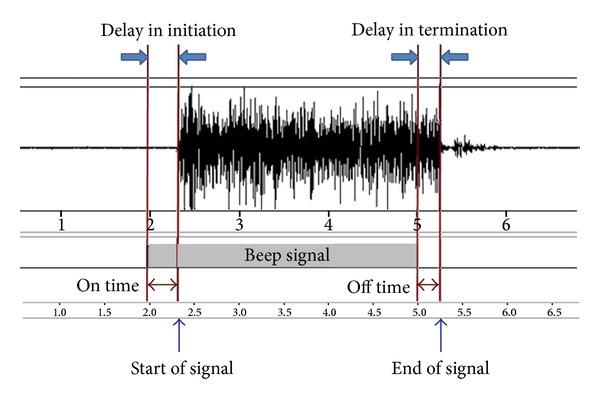
Example of an EMG signal used for RT measurement. The participants were instructed to start and quit the voluntary isometric muscle contraction as quickly as possible when they recognized an auditory beep “on” and “off.” RTi (delay in initiation) was defined as the time interval between the onset of the beep and the onset of EMG contraction signal. RTt (delay in termination) was defined as the time interval between termination of the beep and termination of the EMG signal.

Data acquisition hardware included a 4-channel EMG amplifier QEMG-4 (LMX3204, LAXTHA, KOREA) and National Instruments data acquisition board: PCI 6221 that was interfaced with a personal desktop computer (X-pion TKG X-270, LG electronics, Korea). Data was processed through LabView 8.0 software. The amplifier gain setting was 700, and the bandpass filter frequencies were set to 8~480 Hz. A sampling frequency of 1000 Hz. was used. Two blinded examiners analyzed each tracing of the EMG signal visually and investigated as follows. The beginning and end points were marked manually on the screen. RTi (delay in initiation) was determined as the time interval between the start of an auditory beep and the detection of compound muscle action potential (CMAP) from baseline. RTt (delay in termination) was defined as the time interval between stop of the beep and termination of the EMG muscle activity to the baseline (Figure 2).

### 2.3. Data Analysis

Independent *t*-tests were used to compare basic demographic factors and the chi-square test to evaluate differences in sex ratios between groups. Because PD is basically an asymmetric disorder, RT parameters of the more- and less-affected side were compared using a paired *t*-test. To confirm the relationship between RT with clinical data, Spearman's correlation was used. One-way ANOVA was conducted to compare differences in clinical degree, RT between the three different PD groups classified according to H&Y stage. A *P* value less than 0.05 was considered statistically significant. SPSS version 12.0 for Windows (SPSS Inc, Chicago, IL, USA) was used for all statistical data analyses.

## 3. Results

Both RTi and RTt were significantly greater in the PD group (*n* = 31) than the controls (*n* = 15). Among the 31 PD patients, the right side was more affected in 19 patients, while the left side was more affected in 11 patients. One PD patient could not be categorized as the patient showed symmetric involvement. Both RTi and RTt were significantly greater in the more-affected side than the less-affected side; in particular, RTt was more prominent in the more-affected side (Table 1). Most of the representative motor deficits of PD, that is, bradykinesia, rigidity, and resting tremor, showed significant correlation with RT (Table 2). Both RT values (RTi and RTt) also increased significantly as the sum of the UPDRS part III scores increased. In particular, RT of the less-affected side was more strongly correlated with cardinal motor symptoms and the sum of the UPDRS scores than the more-affected side.

PD patients were categorized into three groups according to their Hoehn and Yahr (H&Y) stage. Seven patients were in Hoehn and Yahr (H&Y) stage 1 (22.6%), 16 were in H & Y stage 2 (51.6%), and 8 had Hoehn and Yahr (H&Y) stage greater than 2.5 (25.8%). Both RTi and RTt were delayed as PD stages advanced (Table 3).

**Figure 3:**
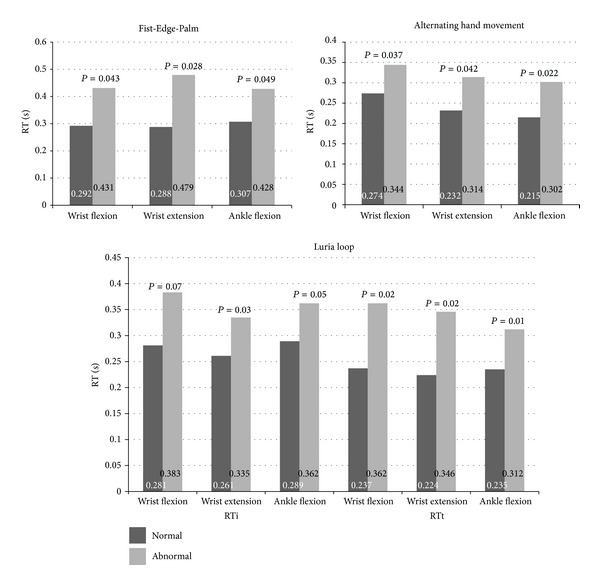
Comparison of the electromyographic reaction time (sec) according to the results of the frontal lobe functions tests.

More than half of the PD patients (52%) were classified as having abnormal frontal lobe function despite most patients being in the early, untreated state of the disease. PD patients with abnormal Fist-Edge-Palm and alternating hand movement tests, reflecting defects in motor programming and motor set-shifting ability, had significantly greater RT values (*P* < 0.05). Patients who had abnormal Luria loop test results, reflecting the presence of perseveration, had also significantly higher RT values than the normal group (*P* < 0.05) (Figure 3). Attention and prefrontal mental set shifting domains were not significantly correlated with RT.

## 4. Discussion

The principal findings of this study are the following: (1) the RT values for both initiation and termination of muscle contraction are significantly longer in PD patients than healthy controls, (2) the degree of RT correlates well with clinically measured motor and cognitive scores in PD patients, (3) RT has some unique features in PD patients (the delay in termination is greater than the delay in initiation and RTt is also more strongly correlated with clinical motor scores than RTi), and (4) RT is longer in the more-affected side and in more-progressed disease states.

### 4.1. Correlation of RT and Clinical Data in Patients with PD

Completion of a motor response requires complex interactions between neural circuits and synaptic connections. In this study, we adopted simple RT tasks that involved one stimulus and one predetermined motor response. The basic processes involved in performing this simple voluntary motor response are signal detection, central signal processing, and motor execution. Delay in muscle contraction of the paretic limbs in stroke survivors has been documented previously [10, 24]. Defects in motor processing may contribute to this delay in stroke patients [10]. The pathophysiology of motor deficits in PD is somewhat different from that in paretic stroke, which involves the pyramidal tract. PD is basically a disease of basal ganglia, which are involved in motor programming and maintenance of motor activity. The motor circuit of the basal ganglia consists of a variety of functional feedback circuits between the basal ganglia and cerebral cortex [25]. Motor deficits in PD are due primarily to failure of the basal ganglia to energize the cortical mechanisms that prepare the muscle for movement [26]. Because of defective basal ganglia function, the cortical networks that enable the corticospinal system to execute voluntary movement fail to adequately control the motor circuit in PD [26]. And Müller et al. [2] previously investigated that the degree of dopaminergic nigrostriatal degeneration measured by [123I]-*β*-CIT SPECT was closely correlated with reaction and movement time in untreated PD subjects. Greater RT in PD patients may be caused by these defective corticobasal ganglia networks. Because PD is a clinically asymmetric disease at least in the early to moderate stages, asymmetric hemispheric dopamine deficiencies exist. Therefore, the finding of greater RT in the more-affected side and more-advanced disease group indicates that the degree of dopamine deficiency may also affect RT. We also demonstrated that PD patients have longer RT that correlated well with clinical features and the severity of the disease. Dopamine acts to inhibit the firing of basal ganglia output nuclei. In the absence of dopamine, excess firing discharge causes inhibition of the thalamocortical motor system, leading to motor slowness [27]. Bradykinesia and rigidity, two cardinal motor manifestations of PD, correlate directly with dopamine level [28]. Although there are controversies, dopaminergic treatment affects reaction and movement time in PD subjects. Mullet et al. demonstrated the findings that simple reaction time delayed after acute levodopa intake attributed to sedative effects to cognitive process and/or dopamine overstimulation [29], while previously dopamine-treated PD group which theoretically tolerated this phenomenon, but not in drug-free group, showed shortened choice reaction time tasks [30]. These findings implicated the possibility of variable clinical impact of dopaminergic treatment on reaction time during acute period. Yanagisawa et al. described how bradykinesia and rigidity in PD lengthen simple RT as well [31]. A previous study demonstrated that, in patients with bradykinesia, the peripheral muscles are not sufficiently energized to perform adequate movements [32], which also supports this bradykinesia-prolonged RT correlation. Another finding that PD patients also take more time to move, because they require a longer time to generate the appropriate forces to move, as reflected by their greater RT in this study [26]. With regard to tremor, dopaminergic neurons of the substantia nigra are indispensable for the development of resting tremor in PD, but the dopamine effect is not always able to explain all of these symptoms [33, 34]. The relatively low correlation between RT and tremor scores can be explained by the fact that resting tremor is not only under direct basal ganglia control but also under the control of related structures such as thalamus, cerebellum, and its connecting systems [35]. Delayed muscle contractions in PD are strongly correlated with the severity of rigidity. Rigidity is also dependent on the central dopamine level, but afferent input from peripheral muscle spindles is essential for the development of rigidity [36]. Because muscular and connective tissue problems may produce rigidity in advanced PD [37], we recruited relatively early-stage patients and excluded those who have peripheral neuropathy. Various degrees of reticulospinal tract involvement in PD result in tonic facilitation of alpha motor neurons [38]. Prolonged RTt might be, at least in part, due to the increased muscle tone resulting from the mechanisms described above. Therefore, the intricate, unfocused, spinal descending pathways, as well as central processing defects, appeared to contribute to the delay in RT. Further studies are required to investigate which anatomical lesions are responsible for this prolongation. RTt was greater than RTi and more highly correlated with clinical scores. If initiation of muscle contraction requires less motor processing than relaxation of contracted muscles, one would expect RTt to be greater than RTi. RTt can be attributed to the characteristic rigidity observed in PD patients, as described above. Another possible explanation of greater RTt is suggested by PD with the phenomenon “paradoxical kinesia;” these patients have difficulty in moving without an external trigger [18, 39]. Although this is not applicable to all PD patients, this phenomenon could explain a more prolonged RTt in response to “signal-off” than initiation response to “signal-on,” a kind of external cue. However, in this study, the response to the auditory cue may have dampened its effect when the participants anticipated a stimuli [40]. However, this paradoxically enhanced movement in response to an externally driven signal could affect the results to a certain degree, because this is not a hallmark of PD but a general property of the motor system that is also adjustable to the normal population [41]. Neurocognitive functions also affect the final motor output. Dysfunction of the neural circuit between the basal ganglia and frontal cortex, resulting from striatal dopamine deficiency, is presumed to contribute to the defects in frontal cognition seen in PD patients. Defected frontal lobe function without dementia in PD is closely related to motor responses. An evoked potential study revealed that the delay in movement initiation in PD might be associated with strengthening of frontal inhibitory systems, which prevent the onset of movement [42]. In another study, consistent with our results, RT correlated well with global cognitive ability, especially, with frontal lobe function, but not attention focusing [14], and impaired frontal lobe function was implicated in a decrease in response speed in PD [3]. In our study, further detailed analysis indicated that subdomains that represent motor set-shifting and motor programming as well as perseveration were more highly correlated with slowed motor responses. Attention, mental set-shifting, and executive functions were less attributable to this motion delay. It is controversial which cognitive components are responsible for motor output in PD, but it is likely that both central cognitive process and motor programming contribute to some extent [43, 44]. In this study, PD patients who had perseveration had significantly delayed motor responses especially in RTt. Based on this finding, we thought that the deficit of response inhibition could also explain why termination was more delayed than initiation [43]. There are some limitations of the study. First, we measured only six times of RT for each muscle. This was because subjects are mostly old aged; they could not bear longstanding isometric contraction for RT measurement, so we decided six times of measure would be enough to see the results. Second, a small number of advanced PD patient were enrolled, due to practical lack of *de novo* advanced PD, and advanced PD patients could not follow the instruction for RT measurement. In this study, untreated, relatively early patients were enrolled to evaluate the fundamental features of PD itself rather than the effects of medication [29, 30]. Widely used and accepted clinical motor rating scales were used to minimize errors and the EMG study was performed immediately after clinical rating to avoid discrepancies due to variable diurnal motor symptoms. Objective quantitative assessments of motor function were made using electromyographic methods. We demonstrated that longer RT is characteristic motor deficits of patients with PD, and RT measurement based on EMG data can serve as a sensitive tool to evaluate motor function in *de novo* PD patients. Both cognition and motor functions are essential for adequate motor responses, but further studies are required to clarify the clinical impacts of these characteristic motor responses.

## Figures and Tables

**Table 1:** Differences in reaction time between Parkinson's disease patients (*n* = 31) and healthy controls based on independent *t*-tests (*n* = 15) and paired *t*-test comparison of the reaction times between the more- and less-affected side for all patients.

		Control (*n* = 15)	PD (*n* = 31)	*P* value	Within PD group (*n* = 30)		*P* value
					More-affected side (Rt 19, Lt 11)	Less-affected side (Rt 11, Lt 19)	
RTi	Wrist flexion	198.8 ± 28.2	264.2 ± 79.9	<0.001**	287.7 ± 93.9	244.1 ± 61.3	0.001**
	Wrist extension	194.9 ± 33.5	253.8 ± 85.7	<0.001**	271.6 ± 100.4	239.0 ± 68.1	0.01**
	Ankle flexion	209.7 ± 27.8	265.0 ± 92.0	<0.001**	284.2 ± 11.8	248.5 ± 55.7	0.021*

RTt	Wrist flexion	242.4 ± 31.6	317.8 ± 75.0	<0.001**	333.0 ± 79.2	303.5 ± 68.7	0.024*
	Wrist extension	236.5 ± 27.2	297.5 ± 63.3	<0.001**	312.9 ± 68.4	280.8 ± 55.9	0.012*
	Ankle flexion	261.8 ± 21.6	306.1 ± 68.4	<0.001**	309.6 ± 78.1	303.6 ± 61.0	0.603

*The mean difference is significant at the 0.05 level.

**The mean difference is significant at the 0.01 level.

Data are presented as mean ± SD (msec).

PD: Parkinson's disease; RTi: reaction time (initiation delay of muscle contraction); RTt: reaction time (termination delay of muscle contraction).

**Table 2:** Spearman's correlation coefficient between EMG reaction time parameters with clinically measured data by UPDRS part III scores.

		Spearman's correlation coefficient between RT and UPDRS part III motor scores					
		More-affected side			Less-affected side		
		Wrist flexor	Wrist extensor	Ankle flexor	Wrist flexor	Wrist extensor	Ankle flexor
RTi	Sum of UPDRS III	0.382*	0.407*	0.440*	0.472**	0.314	0.371*
	Bradykinesia	0.312	0.497*	0.338	0.377*	0.363*	0.215
	Rigidity	0.043	0.120	0.327	0.566**	0.291	0.306
	Tremor	0.367	0.070	0.444*	0.439*	0.351	0.397*

RTt	Sum of UPDRS III	0.392*	0.462**	0.640**	0.524**	0.417*	0.470**
	Bradykinesia	0.428*	0.261	0.530*	0.373*	0.446**	0.375*
	Rigidity	0.383*	0.459*	0.216	0.474**	0.434**	0.375*
	Tremor	0.122	0.103	0.362*	0.142	0.283	0.230

*The mean difference is significant at the 0.05 level.

**The mean difference is significant at the 0.01 level.

UPDRS: Unified Parkinson's Disease Rating Scale; UPDRS part III: sum of motor part score of UPDRS; RTi: reaction time (initiation delay of muscle contraction); RTt: reaction time (termination delay of muscle contraction).

**Table 3:** Comparison of RT values between the three Parkinson's disease groups classified according to Hoehn and Yahr stage.

		Mean latency ± SD (msec)			*P* value
		H&Y stage 1 (*n* = 7, 14 limbs)	H&Y stage 2 (*n* = 16, 32 limbs)	H&Y stage ≥2.5 (*n* = 8, 16 limbs)	
RTi	Wrist flexion	247.9 ± 91.0	241.5 ± 49.2	323.1 ± 94.3	0.040*
	Wrist extension	224.0 ± 36.1	224.5 ± 39.5	338.5 ± 12.3	<0.001**
	Ankle flexion	214.6 ± 23.7	259.3 ± 76.5	320.4 ± 12.7	0.005*

RTt	Wrist flexion	281.8 ± 48.8	310.6 ± 60.2	361.2 ± 99.4	0.058
	Wrist extension	276.2 ± 40.6	285.3 ± 46.3	340.5 ± 88.0	0.001**
	Ankle flexion	289.8 ± 26.8	289.4 ± 53.4	353.8 ± 96.3	0.011*

*The mean difference is significant at the 0.05 level.

**The mean difference is significant at the 0.01 level.

H&Y stage: Hoehn and Yahr stage; RTi: reaction time (initiation delay of muscle contraction); RTt: reaction time (termination delay of muscle contraction); msec: milliseconds.
